# A Pro- and Anti-inflammatory Axis Modulates the Macrophage Circadian Clock

**DOI:** 10.3389/fimmu.2020.00867

**Published:** 2020-05-14

**Authors:** Shan Chen, Kevin K. Fuller, Jay C. Dunlap, Jennifer J. Loros

**Affiliations:** ^1^Department of Molecular and Systems Biology, Geisel School of Medicine at Dartmouth, Hanover, NH, United States; ^2^Department of Biochemistry and Cell Biology, Geisel School of Medicine at Dartmouth, Hanover, NH, United States

**Keywords:** circadian, macrophage, pro-inflammatory, anti-inflammatory, cytokine, PAMPs

## Abstract

The circadian clock broadly governs immune cell function, leading to time-of-day differences in inflammatory responses and subsequently, pathogen clearance. However, the effect of inflammatory signals on circadian machinery is poorly understood. We found that in bone marrow-derived macrophages, some host-derived pro-inflammatory cytokines, e.g., IFN-γ or TNF-α, and pathogen-associated molecular patterns, e.g., LPS or Pam3Csk4, suppress the amplitude in oscillations of circadian negative feedback arm clock components such as PER2, and when examined, specific combinations of these immune-related signals suppressed the amplitude of these oscillations to a greater degree in both bone marrow-derived and peritoneal macrophages. At the transcript level, multiple components of the circadian clock were affected in different ways by pro-inflammatory stimulus, including *Per2* and *Nr1d1*. This suppressive effect on PER2 did not arise from nor correlate with cell death or clock resetting. Suppression of the clock by IFN-γ was dependent on its cognate receptor; however, pharmacological inhibition of the canonical JAK/STAT and MEK pathways did not hinder suppression, suggesting a mechanism involving a non-canonical pathway. In contrast, anti-inflammatory signals such as IL-4 and dexamethasone enhanced the expression of PER2 protein and *Per2* mRNA. Our results suggest that the circadian system in macrophages can differentially respond to pro- and anti-inflammatory signals in their microenvironments.

## Introduction

The solar day/night cycle imposes a 24-h environment on all kingdoms of life, from prokaryotes to humans, and in response, members of all these groups of organisms have evolved ~24-h circadian clocks that can be entrained by the environmental cycles (1). Organisms use circadian rhythms to predict timing of environmental pressures and elicit physiological processes including, in multicellular organisms, time-of-day variations in immune responses (2–5). In mammals, receptors in the retina become activated on light exposure and then transmit information to the suprachiasmatic nucleus (SCN), where the master clock that governs behavior resides (6). Subsequently, the master clock helps to synchronize cells in the periphery (7, 8), including monocytes and macrophages, which exhibit robust cell-autonomous circadian rhythms (2, 9–21). These rhythms are entrained and affected by a range of signals and stimuli, including hormones and nutrients (22, 23); however, many of the important immune signals in circadian biology are uncharacterized.

At the cellular level, the circadian oscillator originates from a transcription-translation feedback loop (TTFL) comprised of several core clock components. A positive arm, consisting of the transcription factors BMAL1 and CLOCK (24–27), drives the expression of clock-controlled genes (CCGs) and also the expression of a negative arm encompassing the protein products of *Per1/Per2/Per3* and *Cry1/Cry2* (28–31). The negative arm represses the activity of the positive arm and thereby closes the feedback loop. An additional interlocking loop is comprised of positive elements encoded by *Rora/Rorb/Rorc* and negative elements encoded by *Nr1d1/Nr1d2* (also known as Rev-erbα/β) (32– 34). This circadian oscillator is cell-autonomous and drives cyclic gene expression in many immune cells including T cells, B cells, macrophages, and more (9, 12, 35–38). Under homeostatic conditions, macrophages rhythmically express ~1,400 CCGs (9), of which a significant portion have immune function (e.g., toll-like receptor expression and others), suggesting that a macrophage can better prime itself in anticipation of pathogen encounter during some times of the day.

During an active infection, pathogen-associated molecular patterns (PAMPs) trigger these receptors on macrophages. Macrophages subsequently release pro-inflammatory cytokines to initiate pathogen clearance. Rhythmic cellular responses may therefore contribute to time-of-day differences in the *in vivo* clearance of microbes, including bacteria (*Salmonella*), viruses (herpesvirus, influenza virus), parasites (*Leishmania*), and fungi (*Aspergillus*) (39–42). However, after the initiation of microbe clearance, little is currently known about how PAMPs or pro-inflammatory cytokines affect the molecular circadian oscillator.

Studies suggest that pro-inflammatory cytokines may influence the clock in several tissue types and model organisms. Repetitive subcutaneous administration of pro-inflammatory cytokines such as interferon (IFN)-α, or to a less well-studied extent IFN-γ, suppresses expression of multiple clock components *Per1/2/3, Clock*, and *Bmal1* mRNA in the mouse SCN (43). Similarly, the presence of IFN-γ in rat SCN cultures suppresses rhythmic PER1^LUC^ protein levels and suppresses neuronal firing rates (44). PAMPs such as lipopolysaccharide (LPS) also appear to suppress *Bmal1* mRNA levels in peritoneal macrophages while other stimuli such as Pam3CSK4 (P3C) do not affect *Bmal1* levels (15). Of interest, recent studies revealed that host inflammatory status affects circadian rhythms: a drug screen targeting inflammation in zebrafish revealed amplitude differences (45), and parasite infection disrupted mouse circadian rhythms (46). In general, the clock responds to pro-inflammatory stimuli, but how the macrophage clock is affected by a range of different inflammatory signals is poorly understood.

To understand how pro- and anti-inflammatory immune modulators might impact the clock in macrophages, we utilized bone marrow-derived macrophages (BMDMs) because they have a robust circadian phenotype and exhibit diversity and plasticity in response to a multitude of activation signals (47, 48). Given that pro-inflammatory signals and anti-inflammatory signals independently act to polarize macrophages toward opposite phenotypes, we hypothesized that the molecular clock in macrophages would respond differently to these stimuli. In this study, we found that some pro-inflammatory signals strongly suppressed PER2 rhythmicity, and that the pathway from IFN-γ to the core oscillator followed a non-canonical pathway from the IFN-γ receptor. We also found that anti-inflammatory signals enhanced the amplitude of PER2 expression, suggesting that the clock responds to different immune-associated stimuli by strengthening or relaxing clock control of gene expression. The results uncover a novel pro- and anti-inflammatory axis in the molecular circadian clock and provide a new perspective on circadian immunobiology.

## Results

### Pro-inflammatory Stimuli Alter PER2 Rhythms in Macrophages

**Figure 1:**
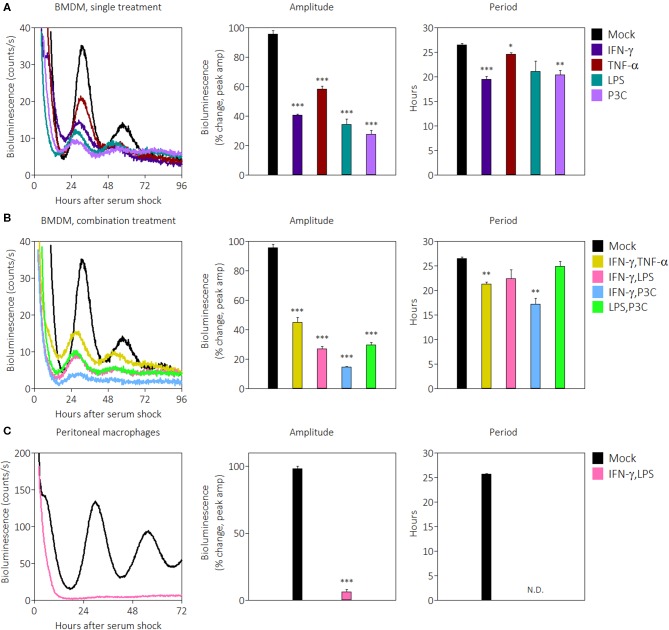
Pro-inflammatory stimuli suppress PER2^LUC^ rhythms in macrophages. **(A)** Raw PER2^LUC^ traces of synchronized m*Per2*^*Luc*^ BMDMs stimulated with mock, IFN-γ, TNF-α, or P3C (50 ng/mL for 24 h), and amplitude and period analysis of rhythms from traces. **(B)** PER2^LUC^ traces of synchronized BMDMs stimulated with mock, IFN-γ plus TNF-α, IFN-γ plus LPS, IFN-γ plus P3C, or LPS plus P3C (50 ng/mL each for 24 h), and amplitude and period analysis of rhythms. **(C)** PER2^LUC^ traces of synchronized peritoneal macrophages stimulated with mock, or IFN-γ plus LPS, and amplitude and period analysis of rhythms. Data are represented as mean ± SE (*n* = 3). *P*-values are calculated from one-way ANOVA with *post-hoc* Tukey tests and are considered to be significantly different with *P < 0.05, **P < 0.005, or ***P < 0.0005.

Previous work demonstrated that systemically administered IFN-γ suppresses *Per1* mRNA levels in the mouse SCN (43). Since IFN-γ is the main cytokine associated with classical activation in macrophages, we investigated the influence of IFN-γ and other pro-inflammatory stimuli on the macrophage clock. The other stimuli included tumor necrosis factor (TNF)-α, a cytokine produced by macrophages, lipopolysaccharide (LPS), a pathogen-associated molecular pattern in gram-negative bacteria, and Pam3CSK4 (P3C), a synthetic triacylated lipopeptide. We generated bone marrow-derived macrophages (BMDMs) from male *mPer2*^*Luc*^ reporter mice, in which the luciferase gene is fused to the endogenous *Per2* gene allowing real-time tracking of PER2 protein (49). The purity of BMDMs was observed by flow cytometry to be over 95% based on cell surface stains for CD11b and F4/80 (Supplemental Figure 1A). After serum-synchronization of BMDMs by FBS, we observed the circadian clock via PER2^LUC^ rhythms. BMDMs in the presence of pro-inflammatory signals exhibited significant reductions in expression of PER2^LUC^ amplitude in the first peak: IFN-γ (40.6% ± 0.9), TNF-α (58.3% ± 2.2), LPS (34.3% ± 3.8), or P3C (27.7% ± 2.7), compared with mock treatments (±SEM; *n* = 3) (Figure 1A). The presence of three signals also shortened period: IFN-γ (19.5 h ± 0.6), TNF-α (24.6 h ± 0.3), and P3C (20.4 h ± 0.9) compared with mock (26.5 h ± 0.3) (Figure 1A). These data indicate that multiple pro-inflammatory stimuli were able to suppress the amplitude and shorten the period of the macrophage circadian clock.

During an active immune response, macrophages may encounter several pro-inflammatory signals *in vivo* simultaneously. In order to identify whether pro-inflammatory stimuli affect the clock additively or whether there is a dominant signal affecting the clock, we combined stimuli at the same concentrations as Figure 1A and observed their effect on PER2^LUC^ rhythms. The results showed that some combinations of pro-inflammatory stimuli suppressed amplitudes to greater extents than their single treatment counterparts. BMDMs in the presence of pro-inflammatory stimuli combinations exhibited reductions in PER2^LUC^ amplitude: IFN-γ plus TNF-α (45.1% ± 3.3), IFN-γ plus LPS (27.0% ± 1.7), IFN-γ plus P3C (14.7% ± 0.2), and LPS plus P3C (29.7% ± 1.9), compared with mock treatment conditions (Figure 1B). The presence of two combinations of stimuli also significantly shortened period: IFN-γ plus TNF-α (21.3 h ± 0.4), and IFN-γ plus P3C (17.2 h ± 1.2), compared with mock treatment conditions (26.5 h ± 0.3) (Figure 1B). These data indicate that a combination of pro-inflammatory stimuli suppressed the amplitude of the clock in an additive manner, dependent on stimulus used. However, this generalization does not extend to period shortening effects. These results suggested the possibility of a dosage effect; therefore, to determine whether increasing IFN-γ dose suppressed amplitude of rhythms to a greater degree, we tested a range of IFN-γ concentrations (from 5 to 200 ng/mL) on BMDMs and observed PER2^LUC^ rhythms. IFN-γ at all concentrations affected PER2^LUC^ rhythms in a similar manner (Supplemental Figure 1C). Similar results were observed when inflammatory signals were given to macrophages and washed out prior to synchronization (data not shown), suggesting the health of the macrophages was not impacted by constant exposure to pro-inflammatory signals. We can conclude that the clock in BMDMs responds to pro-inflammatory stimuli through different pathways to downregulate the expression of circadian clock component PER2. The results, however, do not exclude the possibility that PER2 suppression by pro-inflammatory cytokines is a unique response in BMDMs.

To investigate whether the clock suppressive effect was conserved in other macrophage types, we began by first focusing on the effect of IFN-γ plus LPS. We harvested peritoneal macrophages by thioglycolate elicitation, cultured them overnight, removed non-adherent cells by washing, and then synchronized the population using FBS. The purity of peritoneal macrophages was >80% based on cell surface stains for CD11b and F4/80 (Supplemental Figure 1B). Synchronized peritoneal macrophages were robustly rhythmic as previously reported (9), and in the presence of IFN-γ plus LPS, demonstrated such a remarkable reduction of PER2^LUC^ amplitude (6.2% ± 2.0) that the cells appeared arrhythmic, and therefore the period estimate could not be accurately determined (Figure 1C).

To more systematically determine the effects of singular pro-inflammatory stimuli, PER2^LUC^ rhythms from peritoneal macrophages were observed after treatment with IFN-γ, TNF-α, LPS, or IFN-γ plus LPS (Supplemental Figure 1D). Peritoneal macrophages in the presence of IFN-γ plus LPS again exhibited reductions in PER2^LUC^ amplitude (24.0% ± 2.0) compared with mock (100% ± 17.3) whereas no single signal elicited a significant change in rhythm amplitude: IFN-γ (86.5% ± 11.5), TNF-α (116.7% ± 4.0), or LPS (136.2% ± 20.0) (Supplemental Figure 1D), although LPS alone did increase the overall level of bioluminescence. The presence of IFN-γ plus LPS again promoted arrhythmicity, i.e., no second peak and without an increase in bioluminescence, and IFN-γ alone modestly reduced period (23.5 h ± 0.3) compared with mock (25.7 h ± 0.3) (Supplemental Figure 1D), suggesting that unlike BMDMs, peritoneal macrophages require both activation signals to suppress circadian clock PER2 amplitude. Together, the results indicate that PER2^LUC^ amplitude suppression by some combinations of pro-inflammatory stimuli is not limited to BMDMs and is possibly a pan-macrophage effect.

### Pro-inflammatory Stimuli Alter Core Clock Transcript Rhythms

**Figure 2:**
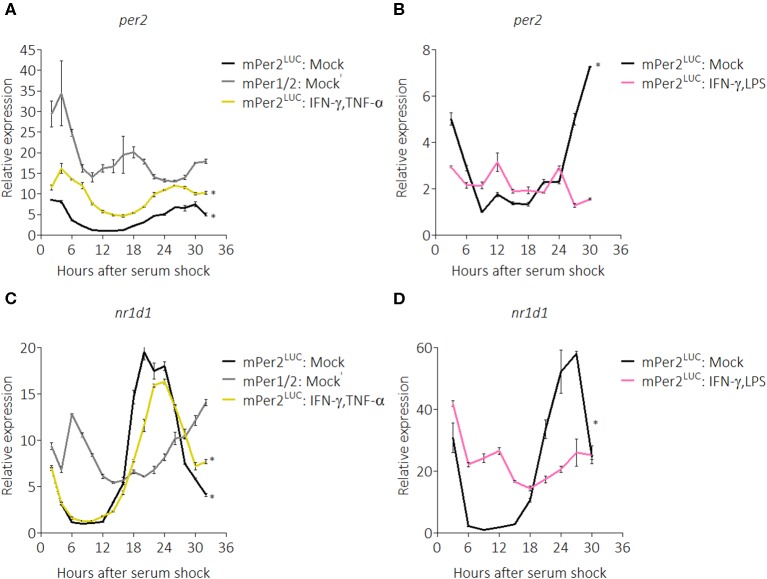
Pro-inflammatory stimuli affect *Per2* and *Nr1d1* mRNA rhythms. qRT-PCR data showing **(A)** *Per2* mRNA expression over a 32 h circadian time course (2 h resolution) from m*Per2*^*Luc*^ BMDMs or m*Per1/2* BMDMs. Cells were stimulated with mock or IFN-γ plus TNF-α (50 ng/mL each for 24 h). **(B)** *Per2* mRNA expression over a 30 h circadian time course (3 h resolution) from m*Per2*^*Luc*^ BMDMs. Cells were stimulated with mock or IFN-γ plus LPS (50 ng/mL each for 24 h). **(C)** *Nr1d1* mRNA expression in exact conditions as in **(A)**. **(D)** *Nr1d1* mRNA expression in exact conditions as in **(B)**. Genes here were normalized to non-rhythmic *Eif2a* expression. Data are represented as mean ± SE (*n* = 3). *P*–values (*) are calculated from Cosinor analysis and significance (at *P* < 0.05) is considered to be rhythmic circadianly.

To ascertain the breadth of the suppressive effect on clock components, we next used quantitative RT-PCR to probe the circadian expression profile of core clock genes in the presence of pro-inflammatory cytokines. We chose IFN-γ plus TNF-α as stimuli because the original characterization of activated macrophages was described in the context of these two cytokines (50). mRNA was harvested from BMDMs over a 32-h time-course with a 2-h resolution and quantified. Gene expression was normalized to a non-circadian housekeeping gene, *Eif2a* (51), and then all data were normalized to mock treatment conditions in *mPer2*^*Luc*^ BMDMs. *mPer1*^*Brdm*1^*mPer2*^*Brdm*1^ (*mPer1/2*) mice harbor deletions of regions in the *Per1* and *Per2* genes essential for proper circadian function (52) and were used as circadian clock-deficient controls. *Per2* mRNA from *mPer2*^*Luc*^ BMDMs exhibited significant circadian rhythms (*p* = 0.0013, robustness = 88.4%, significance determined by a Cosinor regression model) while those from *mPer1/2* were non-circadian (*p* = 1.000, robustness = 22.6%) (Figure 2A). Surprisingly, we found that contrary to the PER2^LUC^ results in Figure 1, *Per2* mRNA levels were elevated in BMDMs stimulated with IFN-γ plus TNF-α (Figure 2A), though, the peak-to-trough amplitude was comparable to mock; these rhythms were determined to be circadian (*p* = 0.0013, robustness = 88.5%), suggesting a role for post-transcriptional control in effecting the cytokine-induced suppression of PER2 protein levels seen in Figure 1.

To investigate whether the same effect on the clock resulted from stimulation with other pro-inflammatory conditions, we performed another time-course (30-h with 3-h resolution) in the presence of IFN-γ plus LPS. We found that in these conditions, *Per2* mRNA levels were suppressed and no longer exhibited circadian rhythmicity (*p* = 1.000, robustness = 11.5%) (Figure 2B). We also explored regulation of the core clock component, *Nr1d1* (also known as *Rev-erb*α), confirming that *Nr1d1* mRNA levels in *mPer1/2* controls did not oscillate, whereas mock-treated *mPer2*^*Luc*^ controls oscillated circadianly (*p* = 0.0005, robustness = 91.8%), as did the IFN-γ plus TNF-α stimulated group (*p* = 0.0003, robustness = 93.5%) (Figure 2C). In contrast, in the IFN-γ plus LPS stimulated group, *Nr1d1* levels were suppressed and lost circadian rhythmicity (*p* = 0.989, robustness = 57.7%) (Figure 2D), suggesting that different inflammatory stimuli impart diverse effects on the clock.

This work was extended to see the effects of pro-inflammatory stimulation by IFN-γ plus TNF-α on expression of other clock components (Supplemental Figure 2). The results revealed that in the presence of IFN-γ plus TNF-α, *Bmal1, Rora*, and *Cry2* mRNA transcripts were differentially expressed and modestly suppressed, despite functional *Per2* and *Nr1d1* rhythmicity (Supplemental Figure 2). Pro-inflammatory stimuli IFN-γ and TNF-α can influence the expression of circadian clock components while maintaining circadian rhythmicity of the cell.

### PER2 Amplitude Suppression Is Not Mediated Through Cell Death or Clock Resetting

**Figure 3:**
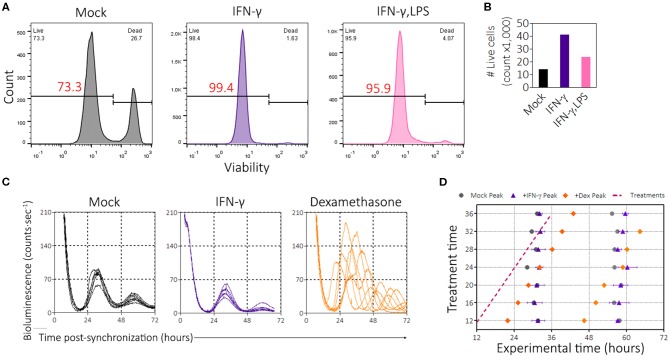
IFN-γ promotes cell survival and proliferation and is not a clock-resetter. **(A)** Histograms of BMDMs stained with viability dye (zombie red, BioLegend). Gating strategy in Supplemental Figure 3A. **(B)** Enumeration of the number of events per group in gated flow plots in **(A)** to determine proliferation. **(C)** Single, averaged, raw bioluminescence traces of BMDMs treated with IFN-γ (50 ng/mL) or dexamethasone (1 μM) between the times of 12 and 36 h, each sample at every 4 h interval. **(D)** Circadian wedge diagram depicting time of treatment (magenta dotted line) and time of resultant peaks from each sample group: mock (black circle), IFN-γ (purple triangle), or Dex (orange diamond). Data are represented as mean ± SE (*n* = 2–3).

In the previous experiments, we noticed that the presence of IFN-γ promoted growth of BMDMs in culture. However, because some pro-inflammatory stimuli such as LPS induce apoptosis (53), we wondered whether the suppression of PER2^LUC^ amplitude by IFN-γ was simply the result of cell death leading to a smaller population of viable cells. To investigate this possibility, we seeded *mPer2*^*Luc*^ BMDMs at a low density (5 × 10^5^ cells per 35 mm dish) to minimize the effect of overcrowding and then treated with inflammatory signals (IFN-γ or IFN-γ plus LPS) for 24 h. The cells were synchronized, and then harvested and stained with a viability dye (Zombie Red, BioLegend) 46 h post-synchronization. Cells were analyzed by flow cytometry, first gating for single cells using FSC-A vs. FSC-H and then by forward- & side-scatter (Figure S3A), then those cell populations were examined for viability (Figure 3A). Mock treatment conditions established a baseline of ~73% live cells (Figure 3A). However, IFN-γ treatment and IFN-γ plus LPS treatments produced cultures that had ~99% and ~96% live cells, respectively (Figure 3A). In culture, IFN-γ treatment resulted in preservation and/or proliferation of BMDMs, as the total number of gated single cells in culture was more than double compared with mock treatment conditions (Figure 3B). The presence of IFN-γ plus LPS stimuli also mildly increased the relative number of cells in culture (Figure 3B). Although BMDMs were seeded at a lower cell density, PER2^LUC^ bioluminescence was still detectable, and suppression by pro-inflammatory stimuli was observed (Supplemental Figure 3). Because IFN-γ can induce metabolic changes in macrophages and because the macrophages were recorded under the LumiCycle in hermetically sealed dishes, we determined that the pH levels in similar cultures were not affected by the presence of IFN-γ after 48 h in culture (Supplemental Figure 3). The results suggest that cell death was not a cause of the reduced PER2^LUC^ signal from the plates. Moreover, PER2 suppression by IFN-γ occurred in spite of an increase in number of cells.

From the limit cycle theory of rhythms, inducing a change in one component of the feedback loop (e.g., a stimulus leading to resetting) can result in a temporarily reduced amplitude of an oscillator such as the circadian clock (54, 55). We therefore wanted to explore the possibility that IFN-γ could reset the clock. To this end, we performed a phase-response experiment in which we treated synchronized *mPer2*^*Luc*^ BMDMs with mock, IFN-γ, or dexamethasone (a known circadian phase-resetter) during live recording of PER2^LUC^ bioluminescence. Treatments were administered between 12 and 36 h after synchronization, every 4 h. Bioluminescence traces showed that dexamethasone enhanced the amplitude and reset the clock (Figure 3C). Consistent with previous results and despite stimulation during the circadian cycle, IFN-γ suppressed the amplitudes of PER2 expression (Figure 3C), suggesting that the mechanism of action was rapid. Peak times were quantified and displayed in a circadian wedge diagram, revealing that IFN-γ did not reset the circadian clock; i.e., the peak expression of PER2 was not set by the time of IFN-γ treatment (Figure 3D). Thus, IFN-γ did not suppress PER2 through clock resetting mechanisms.

### Suppression Is Initiated Through the Interferon Receptor but Is Likely Not Signaled via a Canonical Pathway

**Figure 4:**
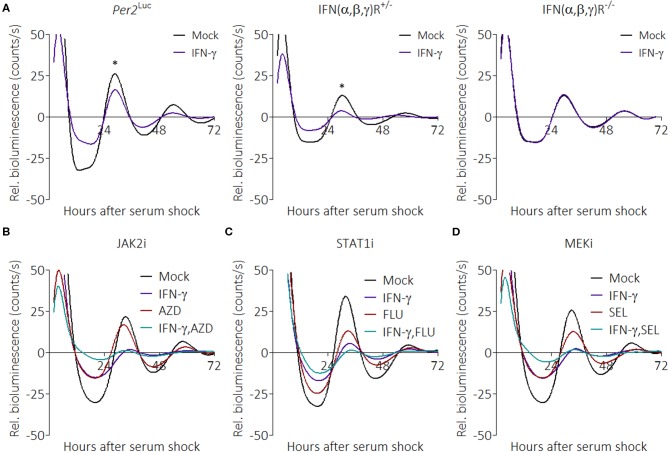
IFN-γ suppresses PER2 through its cognate receptor but does not act through canonical pathways. **(A)** Detrended PER2^LUC^ traces from m*Per2*^*Luc*^, m*Per2*^*Luc*^*IFN(*α*/*β,γ*)R*^+/−^, or m*Per2*^*Luc*^*IFN(*α*/*β,γ*)R*^−/−^ BMDMs treated with mock or IFN-γ (50 ng/mL for 24 h). **(B)** PER2^LUC^ traces from m*Per2*^*Luc*^ BMDMs treated with mock, IFN-γ, JAK2 inhibitor, or IFN-γ plus JAK2 inhibitor. **(C)** PER2^LUC^ traces from m*Per2*^*Luc*^ BMDMs treated with mock, IFN-γ, STAT1 inhibitor, or IFN-γ plus STAT1 inhibitor. **(D)** PER2^LUC^ traces from m*Per2*^*Luc*^ BMDMs treated with mock, IFN-γ, MEK inhibitor, or IFN-γ plus MEK inhibitor. Data are represented as mean (*n* = 3–4). *P*-values (*) are calculated from one-way ANOVA with *post-hoc* Tukey tests at the acrophase and are considered to be significantly different with *P* < 0.05.

Little is known about how pro-inflammatory stimuli affect the circadian clock; therefore, we interrogated the pathways that might be involved. IFN-γ canonically binds to the IFN-γ receptor (IFNGR) to activate inflammatory pathways; however, because IFN-γ can also bind glycosaminoglycan heparan sulfate at the cell surface to induce effects (56), we sought to determine whether the IFN receptor was required for suppression by IFN-γ. To test this, we crossed interferon (α/β,γ) receptor knockout mice to our m*Per2*^*Luc*^ mouse line and generated homozygous and heterozygous offspring [notated *IFN(*α,β,γ*)R*^−/−^ and *IFN(*α,β,γ*)R*^+/−^]. BMDMs were generated from these mice, and synchronized macrophages were treated with IFN-γ. Parental m*Per2*^*Luc*^ BMDMs showed suppressed PER2 rhythms in the presence of IFN-γ, as did *IFN(*α,β,γ*)R*^+/−^ BMDMs (Figure 4A). In contrast, PER2 rhythms were not suppressed in *IFN(*α,β,γ*)R*^−/−^ BMDMs, suggesting the suppression pathway is initiated directly through the IFN receptor.

The canonical IFNGR signaling pathways are JAK/STAT and MEK, therefore we tested their influence on the clock over time with specific inhibitors (57). Synchronized m*Per2*^*Luc*^ BMDMs were given combinations of IFN-γ, AZD1480 (AZD, an ATP-competitive JAK2 inhibitor) (Figure 4B), Fludarabine (FLU, a STAT1 activation inhibitor) (Figure 4C), or Selumetinib (SEL, a highly selective MEK1 and MEK2 inhibitor) (Figure 4D), to see whether inhibition of these pathways would mitigate IFN-γ-mediated suppression of PER2. To our surprise, none of the inhibitors prevented the effect of IFN-γ on the clock. The inhibitors alone were able to influence the clock, suggesting that the results were not due to a deficiency in dosage. These data suggest that suppression of the clock by IFN-γ may occur through one or more non-canonical pathways.

### Anti-inflammatory Stimuli IL-4 and Dexamethasone Enhance PER2 Rhythms

**Figure 5:**
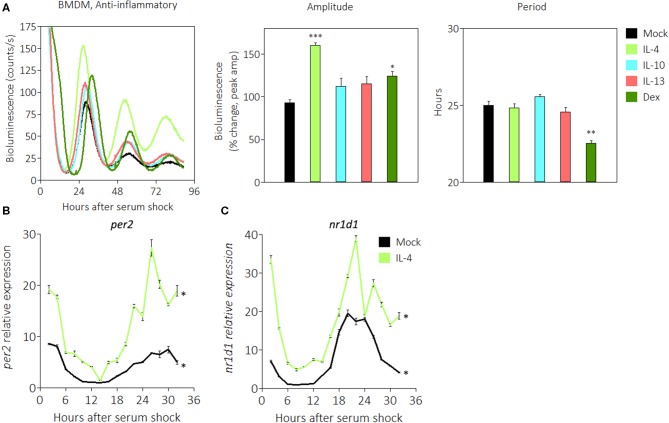
Anti-inflammatory stimuli enhance PER2^LUC^ rhythms in macrophages. **(A)** Detrended PER2^LUC^ traces of synchronized m*Per2*^*Luc*^ BMDMs stimulated with mock, IL-4, IL-10, IL-13, or dexamethasone (Dex), and amplitude and period analysis of rhythms from traces. **(B)** *Per2* mRNA expression over a 32-h circadian time course (2 h resolution) from m*Per2*^*Luc*^ BMDMs. Cells were stimulated with mock or IL-4. **(C)** *Nr1d1* mRNA expression from cells stimulated with mock or IL-4. Data are represented as mean ± SE (*n* = 3). In **(A)**, *P*-values are calculated from one-way ANOVA with *post-hoc* Tukey tests and are considered to be significantly different with *P < 0.05, **P < 0.005, or ***P < 0.0005. In **(B,C)**, *P*–values (*) are calculated from Cosinor analysis and significance (*P* < 0.05) is considered to be rhythmic circadianly.

Macrophage activation can polarize in multiple directions, and anti-inflammatory stimuli skew macrophages toward a phenotype associated with helminth infection or wound healing (48). Glucocorticoids such as dexamethasone (Dex) are anti-inflammatory stimuli that bind to cognate receptors to suppress expression of pro-inflammatory genes (58). We previously observed that PER2 amplitudes were enhanced upon stimulation with Dex (Figure 3C), and this led us to investigate whether other anti-inflammatory signals would similarly affect the circadian clock. Other stimuli tested include IL-4, a cytokine that promotes the alternative activation of macrophages (48), IL-10, a cytokine that inhibits pro-inflammatory cytokine production (59), and IL-13, a cytokine that inhibits pro-inflammatory responses in airway maladies (60). The results revealed that BMDMs in the presence of two anti-inflammatory signals exhibited significant enhancements in PER2^LUC^ amplitude of the first peak: IL-4 (160.0% ± 3.5) or Dex (124.5% ± 8.6), compared with mock treatments (Figure 5A). The presence of only Dex significantly shortened period (22.6 h ± 0.1) compared with mock (25.0 h ± 0.3) (Figure 5A).

We then used quantitative RT-PCR to probe the expression of circadian genes in the presence of anti-inflammatory IL-4. Gene expression was normalized to *Eif2a* and to mock conditions as in Figure 2A. We found that *Per2* mRNA in the presence of IL-4 exhibited significant circadian rhythms, and the expression level was 3–4 fold higher at the acrophase (Figure 5B). Similarly, *Nr1d1* mRNA in the presence of IL-4 exhibited significant circadian rhythms, and the expression level was ~2 fold higher at the acrophase (Figure 5C). These data suggest that some anti-inflammatory stimuli such as IL-4 or Dex act in a manner opposite to pro-inflammatory stimuli to enhance the circadian clock in macrophages.

## Discussion

The ability of macrophages to be activated and polarized into different subsets has been known for several decades (48), yet little is known about how polarization affects the cell autonomous circadian clock. We report here that, in macrophages, pro-inflammatory signals, in general, alter the circadian clock by reducing the amplitude in oscillations of the core clock component PER2, whereas anti-inflammatory signals enhance the amplitude of its oscillations. Although previous studies have shown that inflammatory cytokines could suppress the core circadian clock (43, 44, 61), these studies are largely described in non-immune tissue with the exception of studies showing endotoxin or other PAMPs affecting macrophage clocks (15, 17). We note that we see a strong effect of Pam3CSK4 on the circadian oscillator (Figure 1A) that was not previously observed (15). We also note that the strongly suppressive effect seen with LPS alone in the BMDM circadian clock was not seen in peritoneal macrophages (Supplemental Figure 1D), suggesting unique differences in macrophage clock responsiveness depending on tissue origin. To our knowledge, the work presented here is the first to describe the immune clock's response to pro-inflammatory stimuli such as IFN-γ or immunosuppressive stimuli such as IL-4.

We found evidence for overall perturbation of components of the circadian clock by pro-inflammatory stimuli; notably, in the presence of IFN-γ plus TNF-α, *Per2* mRNA levels had an overall baseline increase (Figure 2A), which was paradoxical in conjunction with our PER2^LUC^ results. However, we note that the fold-change between acrophase and trough in the presence of IFN-γ plus TNF-α was only 3.5 times compared with mock treatment conditions where expression range was 8.5-fold between lowest and highest expression levels. This suggested to us that although *Per2* mRNA was expressing at an overall higher level during pro-inflammatory conditions, the range of oscillations was weaker than in homeostatic conditions. Furthermore, we noted that in the presence of IFN-γ plus TNF-α, *Rora* mRNA appeared to be arrhythmic (Supplemental Figure 2), raising the possibility that the dysfunction of these components of the circadian oscillator may be driving compensatory production of *Per2* mRNA levels, and in turn may be post-transcriptionally regulated to lower protein levels. An emerging caveat is that not all circadianly rhythmic proteins correlate with rhythmic transcripts (62), suggesting the possibility that post-transcriptional regulation may act to integrate metabolic or environmental cues to the clock more rapidly than transcription may allow (63) which may fit in the case of pro- or anti-inflammatory stimuli on the macrophage clock.

In addition to PER2^LUC^ amplitude suppression, macrophages in the presence of IFN-γ proliferate ≥2-fold more compared with mock treatment conditions (Figure 3B). To a lesser extent, the presence of IFN-γ plus LPS also encouraged proliferation (Figure 3B). In pilot experiments, we found that macrophages that were seeded at higher densities before assay in the LumiCycle yielded stronger luciferase signals (data not shown). These results, taken together, were interesting because a larger number of cells per dish ought to have enhanced luciferase signal. This suggested that in Figure 1A, the clock suppressive effect of IFN-γ per cell was even greater than shown. We wondered whether suppression by IFN-γ meant PER2 expression was downregulated in cells or whether cells were simply desynchronizing. Desynchrony would also reduce amplitude because rhythms from separate cells would exhibit destructive interference; however, if cells were desynchronizing, in the presence of IFN-γ, then baseline luciferase signal should rise as time progressed, even as amplitude was reduced. We did not observe this effect in raw, non-detrended bioluminescence traces (Figure 1A), therefore our results exclude the possibility that PER2 suppression is mediated by cell desynchrony.

Because the circadian clock affects around 8% of the transcriptome (i.e., CCGs) (9) and inflammatory cytokines affected expression of the *Bmal1* transcription factor (Supplemental Figure 2), we infer that overall rhythmicity of macrophages is likely affected. During attempts to characterize rhythmic phenotypes of macrophages such as phagocytosis or cytokine secretion in the presence of IFN-γ, we discovered that serum synchronization led to artifacts including quasi-rhythmicity in m*Per1* ^*Brdm*1^*Per2*^*Brdm*1^ macrophages similar to those found in previous studies (20, 64). Some effects may be a consequence of *Per2* mRNA fluctuations in m*Per1/2* BMDMs during 0–8 h after serum shock, seen in Figure 2, and dexamethasone- or forskolin-based synchronization did not resolve these issues. Thus, our *in vitro*-based model may have limitations for determining biological consequences, some of which might be resolved by utilizing *in vivo* studies. However, we note that whole mouse studies also present challenges; for instance, the SCN clock appears to respond to subcutaneous injections of IFN-α or IFN-γ only when these occur at lights-off (ZT12) but not at lights-on (ZT0) (43).

The mechanism of action by pro-inflammatory stimuli on the clock was not elucidated in this study. We approached the problem by first using genetics to ascertain whether the IFN receptor was involved (Figure 4A), then used pharmacological inhibitors to target components of the IFN-γ pathway (Figures 4B–D). We were surprised that, in our hands, none of the targeted components were involved. This suggested to us that an unknown, non-canonical pathway from IFN-γ to the clock was active. During the revision of this manuscript, a study provided evidence that imiquimod, through TLR7, triggers IFN regulatory factor 7 (*Irf7*) in mouse skin and also suppresses several core clock genes (65). Given that multiple pro-inflammatory stimuli were able to suppress PER2^LUC^, it is tantalizing to speculate that there may be a common player, such as *Irf7*, related to all of these stimuli.

Anti-inflammatory signals, especially IL-4 and dexamethasone, enhanced PER2^LUC^ expression. In combination with the pro-inflammatory results, these results are reminiscent of the Th1/Th2 paradigm (66). From this, we suggest that the clock, upon sensing pro- or anti-inflammatory microenvironments, may participate in regulating gene expression profiles (i.e., metabolism, immune-related functions, transmigration, antigen presentation, or wound repair). Macrophages polarized to a pro-inflammatory subset require increased metabolic flux from glycolysis (67) whereas those polarized to an anti-inflammatory subset rely on the TCA cycle and fatty acid oxidation (48, 68). The possibility exists that either the circadian oscillator modulates the amplitude of its rhythms in response to glycolytic flux or that metabolic state of the cell is governed by the robustness of the clock. A recent review highlighted the emergence of interest in immunometabolism and circadian rhythms and the ability to therapeutically target related metabolic pathways (69). It is also tempting to speculate that the host gains flexibility and the ability to re-allocate resources by suppressing circadian clock-related transcription factors in the face of an emerging immunological threat. Our results revealed that the clock in macrophages is affected by diametrically opposing stimuli, and that these stimuli regulate the circadian clock architecture in complex ways. However, it remains to be determined how changes in PER2 amplitude in circadian rhythms affect macrophage biological behavior and circadian output.

## Methods

### Mice

All animal experiments were approved by the Institutional Animal Care and Use Committee at Dartmouth College. Mice were housed in a specific-pathogen-free environment in groups of 4, given *ad libitum* access to food and water, and maintained on a 12:12 light:dark (LD) cycle. We repeatedly backcrossed m*Per2*^*Luc*^ mice and m*Per1* ^*Brdm*1^*Per2*^*Brdm*1^ mice (49, 52) to a C57BL/6J background to >97% homozygosity by SNP mapping. Mice lacking the IFN-α/β and IFN-γ receptors (AG129 or *IFN*α*βγR*^−/−^) in a 129 background (70, 71) were generously provided by Dr. David Leib from Geisel School of Medicine at Dartmouth. *IFN*α*βγR*^−/−^ mice were crossed with m*Per2*^*Luc*^ mice to generate mice heterozygous for both genes, then those were crossed to generate receptor homozygous (m*Per2*^*Luc*^*IFN*α*βγR*^−/−^) and heterozygous (m*Per2*^*Luc*^*IFN*α*βγR*^+/−^) offspring in a mixed background.

### Cell Cultures, Synchronization, and Treatments

Bone marrow-derived macrophages (BMDMs) and peritoneal macrophages were isolated using standard procedures (72). Briefly, bone marrow was flushed from femurs and tibias of 4- to 8-week-old male mice, cells filtered through a 100 μm cell strainer (Fisher), and red blood cells lysed with ACK Lysing Buffer (Gibco) for 2 min. Bone marrow cells were plated at a density of 6 × 10^6^ cells per 35 mm dish, unless otherwise described in the text. Bone marrow cells were then differentiated in culture for 6 days in DMEM (Corning) supplemented with 10% FBS (Gibco), 20% L-929 conditioned medium (LCM), 100 U/mL penicillin/streptomycin (Corning), and 2 mM L-Glutamine (Corning) at 37°C and 5% CO_2_, with a media change on day 3. Peritoneal macrophages were elicited by injecting 3% Brewer Thioglycolate Medium in the peritoneal cavity 4 days before flushing the peritoneum. Resulting macrophages were cultured in DMEM supplemented with 10% FBS, 100 U/mL penicillin/streptomycin, and 2 mM L-Glutamine for at least 4 h before aspirating non-adherent cells and refreshing the media.

To prepare cultures for circadian clock synchronization, cells were first serum-starved for 24 h in Leibovitz's L-15 Medium (Gibco) supplemented with 5% LCM, 100 U/mL penicillin/streptomycin, and 2 mM L-Glutamine. The cells were then synchronized by serum-shock in Leibovitz's L-15 Medium supplemented with 50% FBS for 2 h (73). Subsequently, the cells were cultured in Leibovitz's L-15 Medium supplemented with 5% LCM, 100 U/mL penicillin/streptomycin, 2 mM L-Glutamine, 10 mM HEPES (Gibco), and 100 μM D-Luciferin (GoldBio) for bioluminescence monitoring.

Cultured cells were treated with cytokines, pathogen associated molecular patterns (PAMPs), agonists, or inhibitors. Macrophages were treated with IFN-γ (Peprotech), TNF-α (Peprotech), LPS-EK (InvivoGen), Pam3CSK4 (InvivoGen), and IL-4 (Peprotech), in different combinations at a 50 ng/mL concentration unless otherwise stated. Dexamethasone (Sigma), a glucocorticoid hormone analog that resets the mammalian circadian clock (74), was used at a 1 μM concentration. Inhibitors to multiple biological processes were used: STAT1 activation inhibitor Fludarabine (Selleckchem) at 20 μM, JAK2 inhibitor AZD1480 (Selleckchem) at 5 μM, and MEK inhibitor Selumetinib (Selleckchem) at 10 μM.

For resetting experiments (Figures 3C,D), plates were briefly removed from the LumiCycle and cytokines or glucocorticoids dissolved in DMSO were added at the indicated concentrations at a volume of 2 μL per 2 mL dish. For mock controls, an equivalent amount of DMSO (2 μL) was added. In order to minimize manipulation of the macrophages, the cytokines or glucocorticoids were left on the plates for the entire duration of the experiment. Mock treatments indicated that these manipulations did not themselves reset the clock. In these experiments, because cells were initially synchronized at the outset, treatments whose times span the day but that do not reset the clock will result in phases that align vertically whereas treatments that reset the clock will align diagonally in parallel to the time of treatment across the day.

### Cellular Real-Time Bioluminescence Monitoring and Analysis

Cells harboring *Per2*^*Luc*^ (49) were grown to confluence in a 35 mm diameter tissue culture dish (Corning), and before bioluminescence monitoring, sealed with a 40 mm circular cover glass (Thermo) using silicone high-vacuum grease (Dow Corning) (75). Data were collected in a LumiCycle 32 (ActiMetrics) luminometer for 48–72 h. Raw circadian bioluminescence data were PMT-normalized by subtracting PMT-specific bioluminescence signal without cells to remove baseline signal. Quantification of amplitude was done by subtracting these bioluminescence first peak values by the first trough values in raw data; % change for amplitude was then calculated by normalizing to the max peak value within the mock treatment group. A second order Butterworth filter (baseline-subtraction method in LumiCycle analysis software) and detrending (subtracting luminescence values based on linear regression) was applied to raw luminescence values to minimize noise where indicated, allowing for better period estimates, and were used in several figures for clarity. Period estimates were done by subtracting the time of the second peak from the first peak in rhythms passed through the Butterworth filter.

### RNA Purification, Quantitative RT-PCR, and Primers

Macrophages at different circadian times had supernatants aspirated, and were then frozen immediately at −80°C. Total RNA was then extracted from macrophages using the RNeasy Mini Kit (Qiagen). RNA quality and quantity were assessed by NanoDrop (Thermo), and RNA frozen at −80°C until used. RNA was reverse transcribed, and cDNA was synthesized using the SuperScript III First-Strand Synthesis System (Invitrogen) primed with oligo(dT). cDNA was amplified using iTaq SYBR Green (Bio-Rad) and the StepOnePlus System (Applied Biosystems) using the following primers: *Per2*-F: GCTGCAGTAGTGAGCAGTCT, *Per2*-R: CTCCGCAGGGCATACTTCAA, *Nr1d1*-F: AGGTGGTAGAGTTTGCCAAACAC, *Nr1d1*-R: CACCATCAGCACCTCAAAGGT, *Eif2a*-F: CAACGTGGCAGCCTTACA, *Eif2a*-R: TTTCATGTCATAAAGTTGTAGGTTAGG, *Bmal1*-F: GACCTACTCTCCGGTTCCCT, *Bmal1*-R: GCATATTCTAACTGGTAGTCAGTGG, *Rora*-F: CGCAGCGATGAAAGCTCAAAT, *Rora*-R: CAGGAGTAGGTGGCATTGCT, *Cry2*-F: GCAAGGACTCCTGAGACTGGA, *Cry2*-R: CGTCTGTTGGTGATTGGCTT. *Eif2a* was used as a non-circadian reference gene (51).

### Flow Cytometry

To assess purity of cells, macrophages were dissociated from tissue culture dishes using a non-enzymatic CellStripper Dissociation Reagent (Corning), blocked with anti-mouse CD16/32 (BioLegend), stained with Brilliant Violet 421 anti-mouse F4/80 (BioLegend) and Alexa Fluor 488 anti-mouse/human CD11b (BioLegend) or Zombie Red Fixable Viability Dye (BioLegend), fixed with 4% paraformaldehyde (BioLegend), and analyzed using a MACSQuant VYB (Miltenyi Biotec). Analysis of data was performed using FlowJo software (Becton, Dickson & Company).

### Statistics

Throughout the paper, groups were compared using Student's two-tailed *t*-tests or one-way ANOVA with *post-hoc* Tukey tests. Circadian rhythmicity was determined by the Cosinor method and program (Cosinor Periodogram v3.1) as previously described (76). Statistical analyses were performed using Prism 5 software (GraphPad) or Excel software (Microsoft).

## Data Availability Statement

The datasets generated for this study are available on request to the corresponding author.

## Ethics Statement

The animal study was reviewed and approved by the Institutional Animal Care and Use Committee at Dartmouth College.

## Author Contributions

SC, KF, JD, and JL contributed conception and design of the study. SC performed the statistical analyses and wrote the first draft of the manuscript. All authors contributed to manuscript revision, read and approved the submitted version.

## Conflict of Interest

The authors declare that the research was conducted in the absence of any commercial or financial relationships that could be construed as a potential conflict of interest.
