# Supplementary material for: A Pro- and Anti-inflammatory Axis Modulates the Macrophage Circadian Clock
Source: Front Immunol. 2020 May 14;11:867. doi: 10.3389/fimmu.2020.00867 (PMC7240016; doi:10.3389/fimmu.2020.00867)
Supplement: Supplementary file 1 [file Data_Sheet_1.docx]

A pro- and anti-inflammatory axis modulates the macrophage circadian clock

Supplemental figures and figure captions

# Supplemental Figure 1


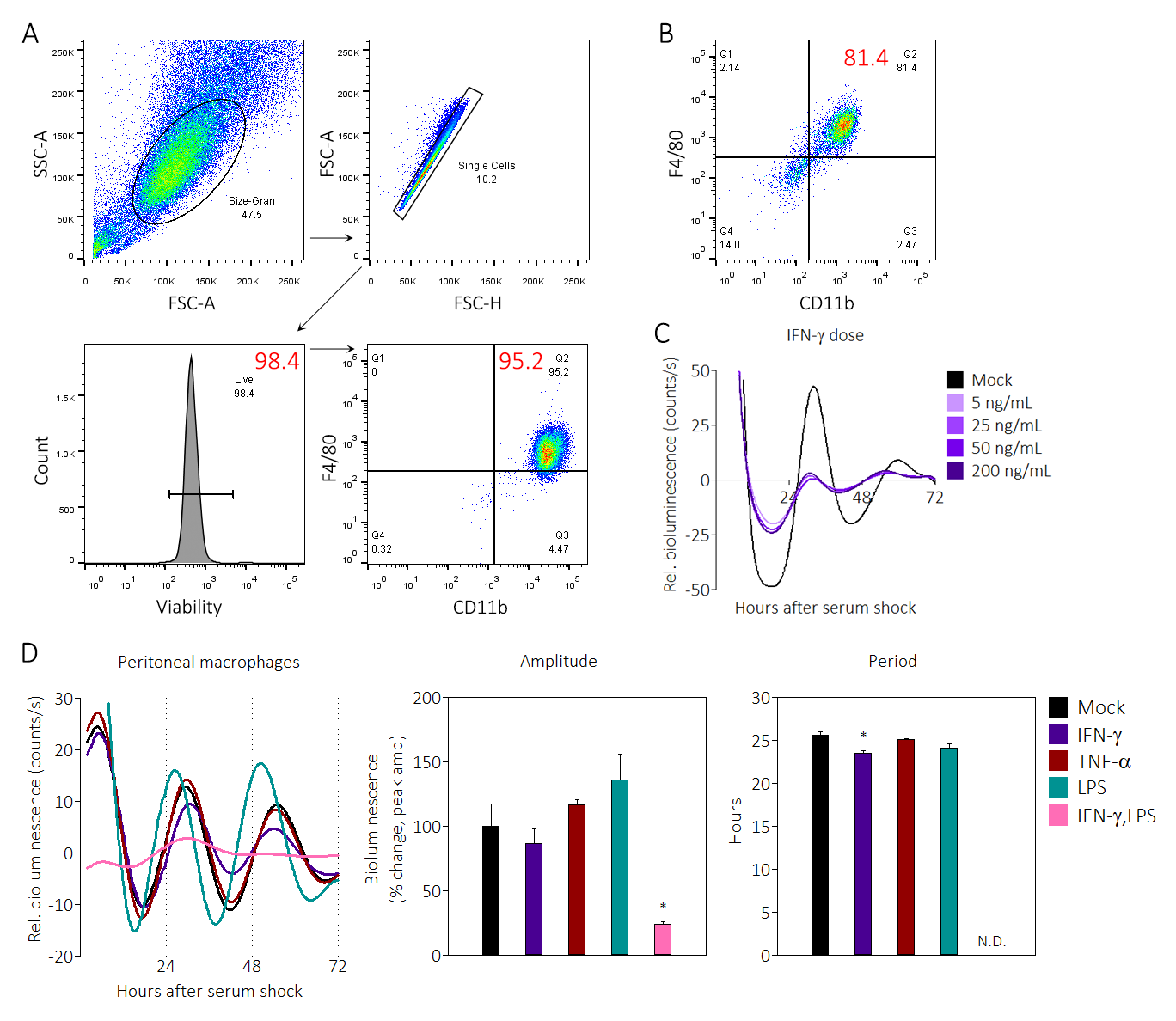


(**A**) Representative flow plots of gating strategy of BMDMs differentiated with L929 supernatant. Cells were gated by side vs. forward scatter, then singlets were selected for by gating cells by forward vs. forward scatter, then viable cells were selected (Zombie Red, BioLegend), and finally cells were quantified by F4/80 and CD11b markers. (**B**) Representative flow plot of peritoneal macrophages and quantification by F4/80 and CD11b. (**C**) Detrended PER2^LUC^ traces of synchronized m*Per2^Luc^* BMDMs stimulated with mock or different doses of IFN-γ. (**D**) Detrended PER2^LUC^ traces of synchronized m*Per2^Luc^* peritoneal macrophages stimulated with mock, IFN-γ, TNF-α, LPS, or IFN-γ plus LPS (50 ng/mL each for 24 h), and amplitude and period analysis of rhythms from traces.

# Supplemental Figure 2


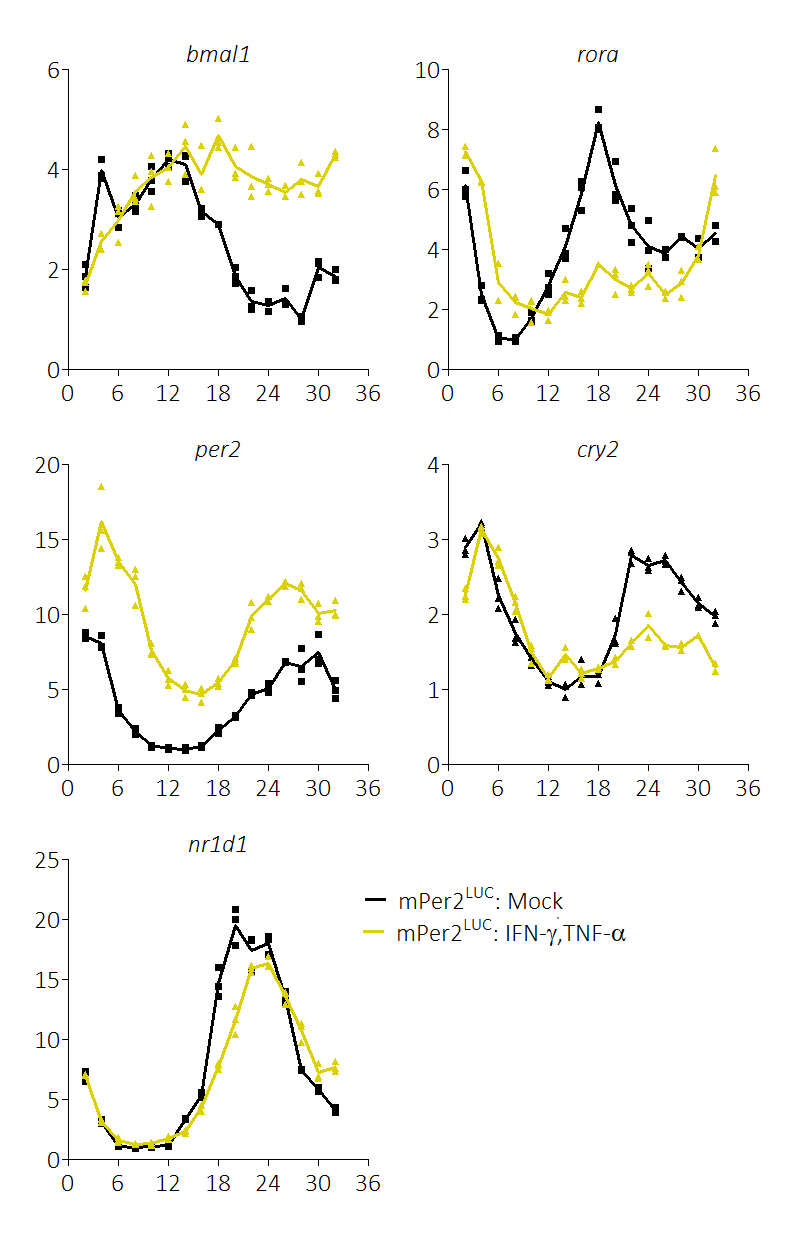


mRNA expression of circadian clock components (*Bmal1*, *Rora*, *Per2,* *Cry2*, and *Nr1d1*) over a 32-h circadian time course (2 h resolution) from m*Per2^Luc^* BMDMs stimulated with IFN-γ plus TNF-α (50 ng/mL for 24 h). Data are represented as mean (n=2-3).

# Supplemental Figure 3


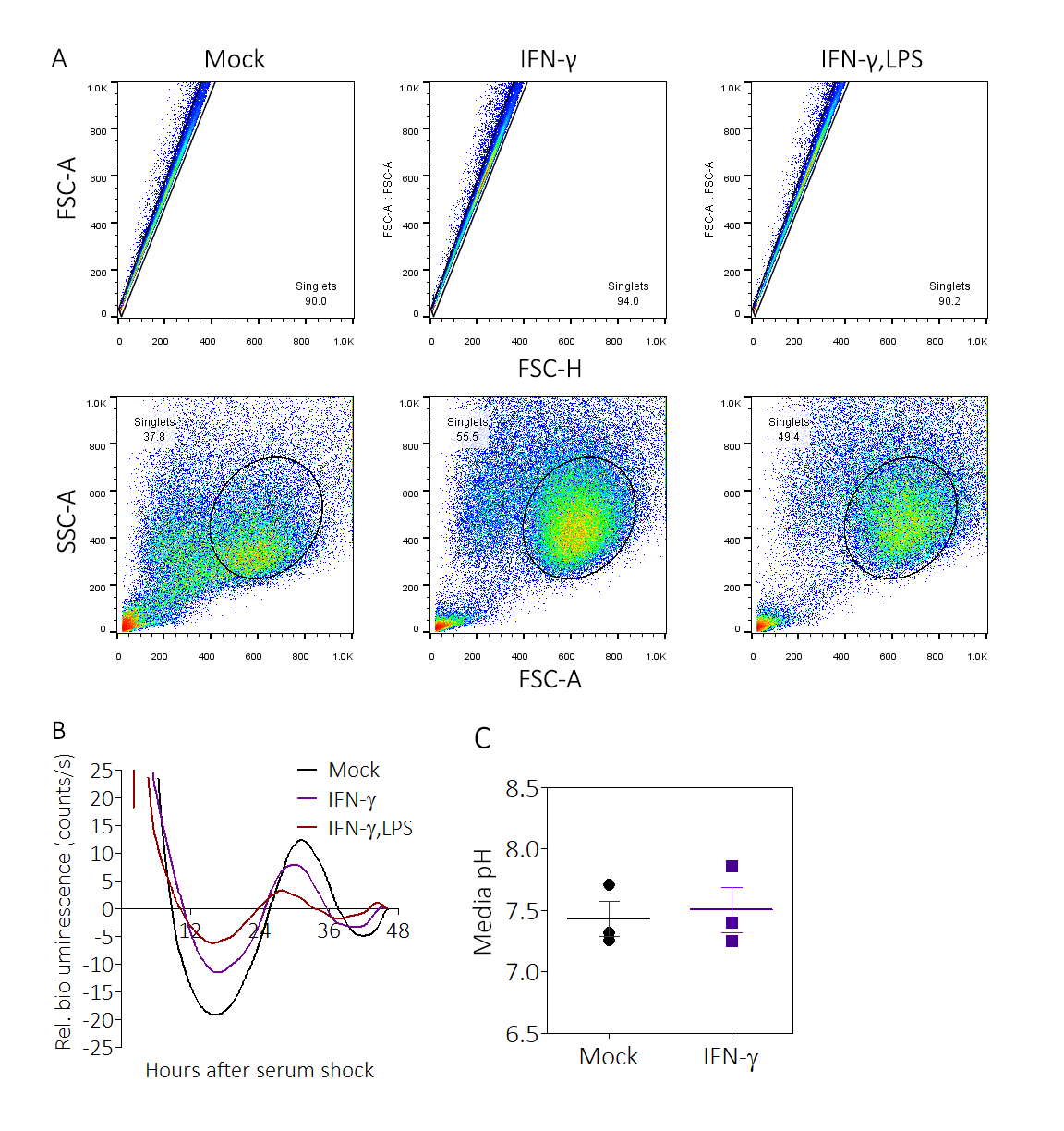


(**A**) Flow plots of BMDMs in response to IFN-γ or IFN-γ plus LPS stimulation (50 ng/mL each for 24 h) and gating strategy for selecting single cells (FSC-A versus FSC-H, then by SSC-A versus FSC-A) which were then analyzed for viability in Figure 3A. (**B**) Detrended PER2^LUC^ traces of synchronized m*Per2^Luc^* BMDMs (seeded at low density) stimulated with mock, IFN-γ, or IFN-γ plus LPS. (**C**) pH of supernatant of synchronized BMDMs after 48 hours in culture.
